# Prefusion structure of human cytomegalovirus glycoprotein B and structural basis for membrane fusion

**DOI:** 10.1126/sciadv.abf3178

**Published:** 2021-03-05

**Authors:** Yuhang Liu, Kyle P. Heim, Ye Che, Xiaoyuan Chi, Xiayang Qiu, Seungil Han, Philip R. Dormitzer, Xinzhen Yang

**Affiliations:** 1 Discovery Sciences, Pfizer Inc., Groton, CT 06340, USA.; 2 Vaccine Research and Development, Pfizer Inc., Pearl River, NY 10965, USA.

## Abstract

Human cytomegalovirus (HCMV) causes congenital disease with long-term morbidity. HCMV glycoprotein B (gB) transitions irreversibly from a metastable prefusion to a stable postfusion conformation to fuse the viral envelope with a host cell membrane during entry. We stabilized prefusion gB on the virion with a fusion inhibitor and a chemical cross-linker, extracted and purified it, and then determined its structure to 3.6-Å resolution by electron cryomicroscopy. Our results revealed the structural rearrangements that mediate membrane fusion and details of the interactions among the fusion loops, the membrane-proximal region, transmembrane domain, and bound fusion inhibitor that stabilized gB in the prefusion state. The structure rationalizes known gB antigenic sites. By analogy to successful vaccine antigen engineering approaches for other viral pathogens, the high-resolution prefusion gB structure provides a basis to develop stabilized prefusion gB HCMV vaccine antigens.

## Teaser

Prefusion human cytomegalovirus glycoprotein B structure reveals the structural basis for membrane fusion.

## INTRODUCTION

Human cytomegalovirus (HCMV) is a leading cause of deafness and developmental delay following transmission in utero (*1*). It also causes opportunistic disease in immunocompromised hosts and lifelong latent infections. Developing a congenital HCMV vaccine is a public health priority and a challenge due to a need for longer duration and more complete protection than has been demonstrated with vaccine candidates tested clinically to date (*2*–*4*). Antibodies directed against the viral fusogen, glycoprotein B (gB), can neutralize HCMV (*5*). The irreversible transition of gB from a prefusion to a postfusion conformation mediates fusion of the HCMV envelope with a cellular membrane during entry (*6*–*8*). For some viruses, such as respiratory syncytial virus (RSV), immunization with the viral fusogen in its prefusion conformation has proven more capable of eliciting neutralizing antibodies than immunization with the fusogen in its postfusion conformation (*9*). However, prefusion gB conformational instability has prevented its use as a vaccine antigen or as a target for high-resolution structure determination.

High-resolution structures of herpesvirus postfusion gBs reveal a conserved arrangement of domains, despite only 24.2 and 30.2% amino acid identity between HCMV gB and its herpes simplex virus 1 (HSV-1) and Epstein-Barr virus (EBV) homologs, respectively (*7*, *8*, *10*, *11*). The postfusion structure of an RNA virus fusogen, vesicular stomatitis virus (VSV) G, reveals an unexpected similarity to postfusion gBs of herpesvirus, which are DNA viruses, despite the lack of amino acid sequence similarity (*12*). Unlike herpesvirus gBs, VSV G is in a dynamic equilibrium between prefusion and postfusion conformations and stable enough at alkaline pH in the prefusion conformation for structure determination (*12*). A high-resolution structure of prefusion VSV G reveals a tripod-like architecture, with exposed fusion loops pointing toward the anchoring membrane (*13*). High-resolution herpesvirus gB prefusion structures have been elusive, although ~9- to 20-Å-resolution electron cryotomography (cryo-ET) molecular envelopes of surface glycoproteins on HSV-1 and CMV virions and of a mutation-stabilized prefusion HSV-1 gB on extracellular vesicles indicate that prefusion herpesvirus gBs also have tripod-like structures and an arrangement of domains like that of VSV G (*6*, *14*, *15*).

## RESULTS

### Prefusion gB stabilization and structure determination

The determination of previous high-resolution prefusion fusogen structures was enabled by naturally occurring stable prefusion states of influenza hemagglutinin (*16*) and VSV G (*13*) and by stabilization with a bound prefusion-specific monoclonal antibody (mAb) fragment for RSV F (*17*). Because such approaches were not feasible for HCMV gB, we stabilized prefusion gB in the viral envelope by adding a thiourea fusion inhibitor, WAY-174865 (fig. S1A) (*18*–*20*), during virus and protein purification and chemically cross-linked virions with bis(sulfosuccinimidyl) glutarate (BS^2^G). Cross-linked gB was bound with an affinity-tagged fragment of a human-derived HCMV neutralizing mAb, SM5-1 (*21*), extracted from virions with detergent, and purified by affinity and size exclusion chromatography (fig. S1B).

**Fig. 1:**
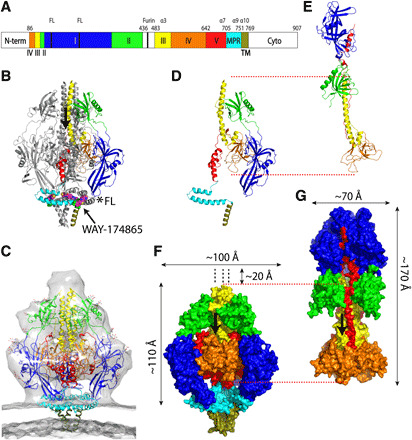
Key structural features. (**A**) Linear representation of HCMV gB (Towne strain) domains labeled and color-coded as follows: I, blue; II, green; III, yellow; IV, orange; V, red; membrane-proximal region (MPR), cyan; transmembrane (TM), olive; unmodeled N-terminal residues (N-term), residues 436 to 483, and cytoplasmic residues (Cyto), white. The boundary amino acid numbers are indicated at the top. The fusion loops (FL), furin cleavage site, and key α helices are indicated. Domain color scheme is maintained in all panels. (**B**) Ribbon diagram of prefusion gB with space-filling model of the bound inhibitor WAY-174865 (magenta). In one protomer, the domains are colored, and the fusion loops (FLs) are indicated with an asterisk. (**C**) Prefusion gB ribbon diagram fit to the cryo-ET density map of the CMV viron surface (EMD-9328) (*6*). Protomers of prefusion and postfusion gB are compared in (**D**) and (**E**). The dimensions of the trimeric ectodomains are indicated on space-filling models of prefusion (**F**) and postfusion (**G**) gB. The approximate height of an unbuildable membrane-distal part of the density map of prefusion gB is indicated. Red dashed lines connect equivalent positions of domains III and IV of the prefusion and postfusion gB structures in (D) and (E) and in (F) and (G). In (B), (F), and (G), the N- to C-terminal direction of the domain III coiled coil is indicated by vertical arrows.

Classification of electron cryomicroscopy (cryo-EM) images of the preparation revealed one class that contained approximately 55% of the molecules and was identifiable as postfusion gB (*7*, *8*, *10*, *11*). A second class that contained approximately 13% of the molecules had distinct protein features but did not resemble the known structure of postfusion gB, and the remainder lacked distinct protein features (fig. S2A). Three-dimensional (3D) image reconstructions yielded two distinct structural maps—a 3.5-Å-resolution map of a postfusion gB–SM5-1 Fab complex and a 3.6-Å-resolution map of a prefusion gB–SM5-1 complex, including the detergent micelle around the transmembrane (TM) domain (fig. S2, A to C, and table S1). No intermediate forms between prefusion and postfusion were detected. The prefusion gB density map allowed model building for residues 86 to 436 and 483 to 769 (Fig. 1, A and B, and fig. S3); the postfusion gB density map allowed model building for residues 87 to 113, 120 to 439, and 474 to 697 (Fig. 1, D to G). The molecular model of prefusion gB fits well in the low-resolution electron cryo-ET envelope of prefusion gB on the virion (*6*) (Fig. 1C), confirming that the structure represents a prefusion conformation and that the stabilization of the prefusion gB by the fusion inhibitor and chemical cross-linker did not substantially distort its overall structure.

### Prefusion gB structure

In the model, prefusion gB forms a tripod that is approximately 100 Å in diameter and extends 110 Å above the viral membrane. Another 15 to 20 Å of height is added by a membrane-distal knob that is apparent from mass density but insufficiently resolved for reliable model building (Fig. 1F and fig. S2C). In contrast, postfusion gB, which was reconstructed from another class of particles in the preparation and from published structures (*7*, *8*), is much narrower (70 Å diameter) and taller (170 Å above the fused viral and cell membrane; Fig. 1G and fig. S2C). This notable difference between the prefusion and postfusion structures indicates a major rearrangement accompanying membrane fusion.

The central core of the ectodomain is formed by three trimeric coiled coils centered on the threefold axis with an N- to C-terminal polarity toward the center of the virion: one membrane distal in domain III (α3, yellow), one membrane proximal in domain V (α7, red), and the TM domain, which is embedded in the viral envelope (α10, olive; Figs. 1, B, D, and F, and 2). There is no indication that the N-terminal part of the domain III α3 helix diverges from the threefold axis, as suggested in a model of an engineered HSV prefusion gB based on a ~9-Å cryo-ET structure (*15*). The membrane-distal part of the globular domain IV (orange), which is primarily composed of β sheets and coils, packs against the domain III central coiled coil (α3). The membrane-proximal end of domain IV diverges from the threefold axis, making a vault-like cavity that accommodates part of domain V (red; Fig. 3A). The N-terminal part of domain V between residues 642 and 660 is sequestered from solvent (Fig. 3A). The C-terminal part of domain V (α7, residues 683 to 704), which forms the membrane-proximal ectodomain coiled coil with its counterpart in the other protomers, does not connect directly to the TM domain. Rather, in this type III fusogen, C-terminal to domain V, the peptide backbone of each protomer diverges from the threefold axis to form the two glycine-rich amphipathic helices (α8 and α9) of the membrane-proximal region (MPR), which may be involved in the fusion process (Fig. 3B) (*22*, *23*). These two MPR helices form a hairpin in the plane of the membrane, likely at the outer leaflet between the phosphate head groups and acyl chains, with the membrane-facing side composed primarily of hydrophobic residues, and the exterior-facing side composed primarily of polar residues. In the trimer, the three MPRs form a ring around the threefold axis (Fig. 4C and fig. S4E).

**Fig. 2:**
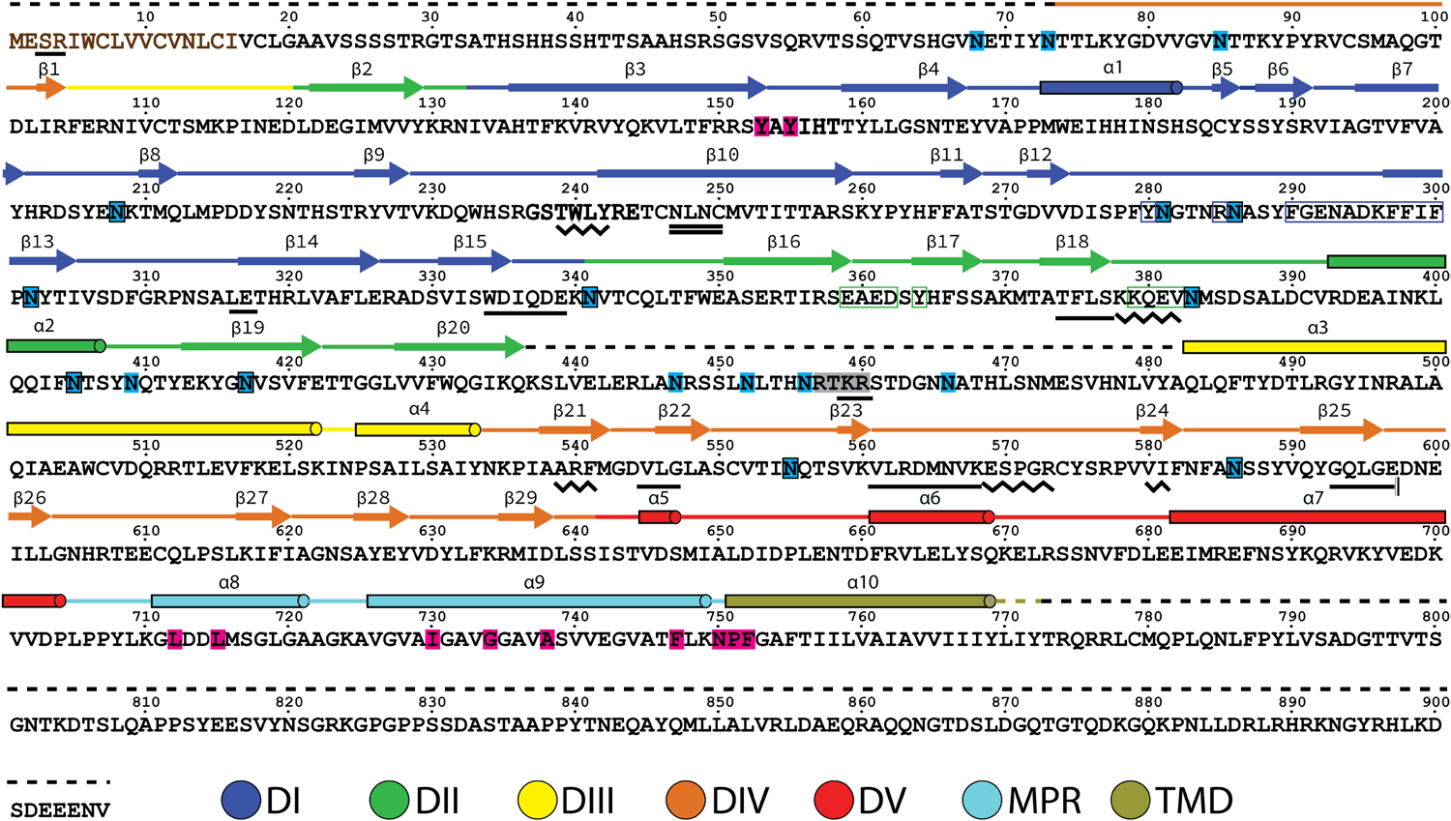
Structural features of gB mapped to the primary amino acid sequence. Secondary structural elements of the prefusion structure (Towne strain) are color-coded as in Fig. 1A and indicated above the sequence: α helices, cylinders; β strands, thick arrows; loops, solid lines; residues missing from the model, dashed lines. Postfusion secondary structural elements that differ from the prefusion structural elements are indicated above the prefusion elements in black: α helices, waved lines; β strands, double lines; loops, straight lines. Features of the primary amino acid sequence are indicated by the color of the letters or colored outline or solid boxes around the letters: secretion signal sequence, brown bold letters; fusion loops, black bold letters; predicted N-linked glycosylation sites, solid light blue boxes; glycosylation sites observed in the density map, solid light blue boxes with black outlines; residues that contact the fusion inhibitor, solid magenta boxes; furin cleavage site, gray solid boxes; residues that contact antigenic domain 4 FAb SM5-1 in the complex, green outline boxes; escape mutations for antigenic domain 5 mAbs 1G2 (Y280 and N284) and 2C2 (N293 and Y280), blue outline boxes.

**Fig. 3:**
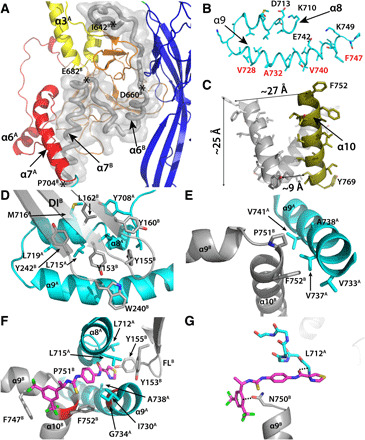
Structural details of prefusion gB. When multiple protomers are shown, they are labeled with superscripts. (**A**) Domain V interprotomer interactions. Ribbon diagrams of domains from one protomer are colored as in Fig. 1A. Backbone of domain V of an adjacent protomer is a gray tube in a semitransparent gray space-filling model. Helices α3, α6, and α7 are labeled. Key residues described in the main text are indicated. (**B**) MPR structure. Representative solvent-exposed hydrophilic residues are labeled with black type, and representative hydrophobic residues that interact with the membrane are labeled with red type. (**C**) TM domain structure. Residues and dimensions described in the main text are indicated. (**D**) Interaction between the MPR and the fusion loop of domain I from an adjacent protomer. (**E**) Interaction of the MPR of one protomer with the hinge between the MPR and TM of an adjacent protomer. (**F**) Hydrophobic interactions of the inhibitor and gB. Inhibition escape mutation locations are red. (**G**) Polar interactions between the inhibitor and gB. Hydrogen bonds and dipole interactions described in the text are indicated with dashed lines.

**Fig. 4:**
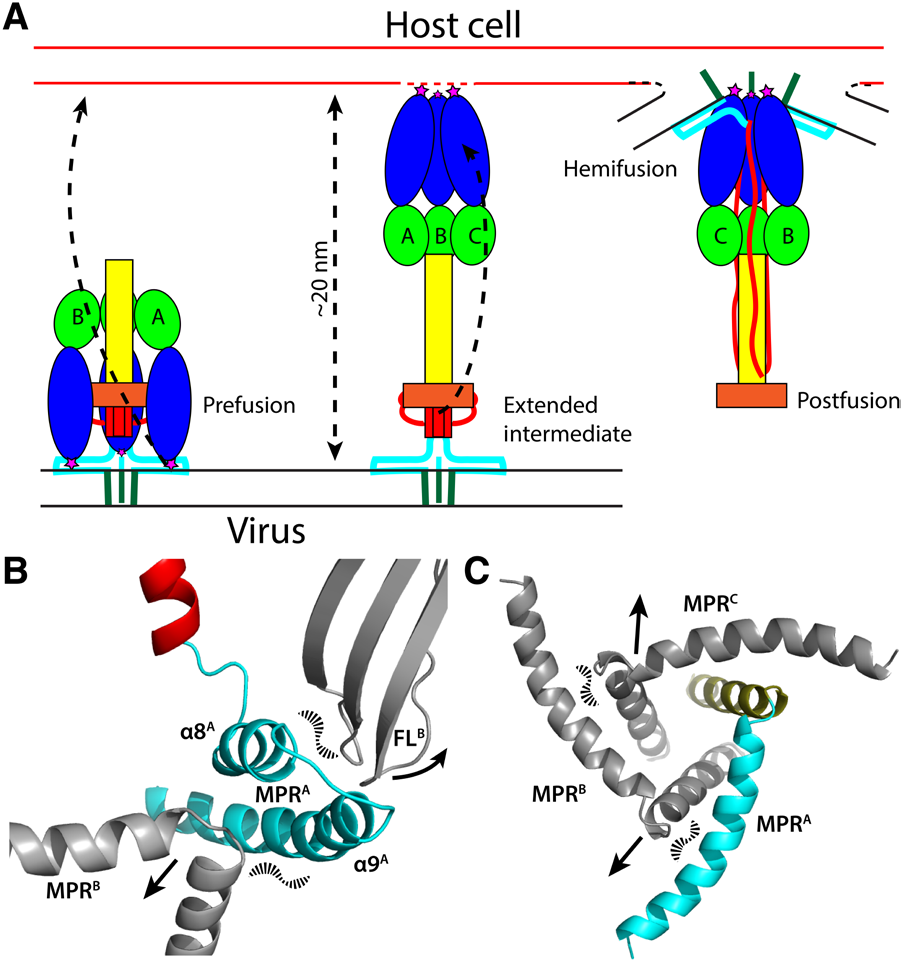
Model of gB rearrangement. (**A**) In prefusion gB (left), the fusion loops (purple star) interacts with the MPR (cyan), embedded in the viral envelope’s (black parallel lines) outer leaflet. To form the hypothesized extended intermediate (middle), fusion loop interactions with the MPR are broken, and domains I (blue) and II (green) rotate almost 180° (left dashed arrow) so that the fusion loop, now at the viral envelope–distal end of gB, interacts with the host cell membrane (red parallel lines), bridging the viral envelope to the host cell membrane. Domain V (red), which had been covered by domain I, is exposed. During the transition from the extended intermediate to postfusion gB (right model), domain V extends toward the cell membrane (middle dashed arrow), packing between domain I from the other two protomers. The MPR and TM (olive) are juxtaposed, facilitating formation of the hemifusion intermediate between the viral envelope and host cell membrane, which resolves to complete fusion. (**B**) Breaking of the interactions between the fusion loops of one protomer with the MPR/TM hinge of an adjacent protomer during the prefusion to extended intermediate transition. (**C**) Movement of MPRs away from the threefold axis during the extended intermediate to postfusion transition.

C-terminal to the MPRs and separated from them by Pro^751^, the single TM helix (α10) of each protomer angles toward the threefold axis (Fig. 3C), with Phe^752^ at the exterior end separated by ~27 Å between protomers, and the interior residue closest to the axis, Ile^768^, separated by ~9 Å. The distance along the threefold axis from the external end (Phe^752^) to the most interior resolved residue (Tyr^769^) is ~25 Å, shorter than the usual ~30 Å of TM domains (*24*, *25*), suggesting that the HCMV gB TM extends inward beyond buildable density (Fig. 3C). The TM coiled coil of postfusion HSV gB converges more steeply, from ~26 Å of separation at the exterior end (Phe^775^) to ~4 Å at its narrowest point (Ala^791^) before diverging again at the interior end (fig. S4E) (*24*). The limited interprotomer interactions in the TM domain from the prefusion HCMV gB model suggest that the tilting angle of the TM helices will be determined mainly by the cytoplasmic tail domain, which may be able to perturb the lipid membrane independent of the ectodomain and is not resolved in high-resolution HCMV gB prefusion or postfusion structures.

The legs of the prefusion trimer are formed by the peripheral, globular domain I of each protomer, which has the fusion loops at its membrane-proximal tip (Fig. 1, B to D and F, blue). Domain I is linked to the more membrane-distal, peripheral, globular domain II (green), which packs tightly against the central helix of domain III (α3). Because domains I and II are only tethered to the central core at the membrane-distal knob of the molecule (Fig. 1, B to D and F), there is potential for them to be mobile relative to the core. The tether between domains II and III (yellow) contains the activating protease cleavage site and is not in buildable density between residues Q436 and Q483 in the cryo-EM image reconstruction (fig. S5A).

### Structural rearrangements during membrane fusion

Comparison of the prefusion and postfusion gB structures shows that domains I to IV rearrange extensively relative to each other, but each of these domains largely retains its fold (Figs. 1, D to G, and 4A and fig. S4). Relative to the central core consisting of the long domain III coiled coil (α3 helix) with the globular domain IV at its C-terminal end, rearrangements at the hinge between domains II and III enable domains I and II of each protomer to flip upward as one unit (Fig. 4A), rotating ~180° into its postfusion position. The hydrophobic fusion loops at the tips of domain I, which had pointed parallel to the N to C polarity of the central domain III coiled coil, now point antiparallel to the coiled coil. We hypothesize that, similar to other viral fusogens (*26*), CMV gB transitions through an extended intermediate, in which gB connects the virion envelope and a host cell membrane and bridges an approximately 20-nm gap between the membranes (Fig. 4A).

To transition from the extended intermediate to the postfusion conformation, domain V, buried in the interior of the prefusion structure as a series of α helices separated by loops (Fig. 3A), refolds. Several domain V prefusion α helices (α5, α6, and part of α7; Fig. 2) become extended coils, as domain V rearranges to its postfusion structure, which is external and extended, packing into the grooves between the α helices of the central domain III coiled coil and the grooves between the rotated domains I and II from adjacent protomers (Figs. 1G and 4A) (*8*). The refolding of domain V brings the MPR to which it is attached and the TM region to the same end of the molecule as the fusion loops so that the host cell membrane (into which the fusion loops are inserted) and the virion envelope (into which the TM coiled coil is inserted and the MPR amphipathic helices are embedded) are brought together, leading to membrane fusion and viral entry. Domain IV, which had been largely buried at the center of the prefusion trimer, becomes an exposed “crown” at the end of the postfusion trimer distal to the fused viral and cell membranes (Fig. 4A). This is a variant on the progression of conformational changes familiar from other well-studied fusion proteins (*26*).

### The membrane-proximal region

The interactions that hold prefusion gB in its metastable prefusion state until triggered by an unknown event, potentially involving interactions with the CMV envelope glycoproteins gH/gL during viral entry (*27*, *28*), are apparent from the structure. In the prefusion conformation, domain V residues 639 to 669 from each protomer are sandwiched between domains I and IV of an adjacent protomer, “tacking” domain I down to the central core (Fig. 3A). Domain I is further constrained by the interaction of multiple aromatic side chains (Y153, Y155, Y160, W240, and Y242) in its two fusion loops (between β3 and β4 and between β9 and β10; Fig. 2) with the amphipathic α8 and α9 helices of an adjacent protomer’s MPR, which forms a “scabbard,” shielding the hydrophobic tip of domain I until it is unsheathed to insert into a host cell membrane in the extended intermediate (Figs. 3D and 4, A and B). This shielding could prevent the conserved fusion loops from inserting fully in the virion envelope and from being bound by antibodies. The aromatic rings of fusion loop residue W240 may also interact with the outer leaflet of the viral envelope (Fig. 3D). Hydrophobic interactions of a set of valine side chains (V733, V738, and V741) on one face of the MPR α9 helix with F752 in the TM and P751 in the loop between the MPR and TM of an adjacent protomer must be disrupted as the MPR trimeric ring separates to allow the refolding and extension of domain V needed for the extended intermediate to transition to the postfusion conformation (Figs. 3E and 4).

The MPR and TM region were largely disordered in the postfusion HCMV gB structure, which prevented a direct comparison of this region in prefusion and postfusion HCMV gB. However, in the previously determined HSV postfusion gB structure, a single MPR amphipathic helix, in the plane of the membrane, immediately N-terminal to the TM domain, and without a buildable connection to the remainder of the ectodomain, was resolved (fig. S4D) (*24*). This HSV gB MPR amphipathic α helix is equivalent to the C-terminal α9 helix of the HCMV gB MPR. The resemblance of the prefusion HCMV gB MPR and TM regions to the resolved part of the postfusion HSV gB MPR and TM regions suggests that the HCMV MPR and TM region form similar structures in the prefusion and postfusion conformations. The single modeled HSV postfusion gB MPR α helix does not interact with the HSV gB fusion loops, in contrast to the direct scabbard-like interaction of the prefusion HCMV gB MPR with the HCMV gB domain I fusion loops (Fig. 3, D and F, and fig. S4D). This difference could reflect missing MPR buildable density in the HSV postfusion gB structure, a distinction between HSV gB and HCMV gB, an interaction in prefusion HCMV gB that is dependent on the presence of the fusion inhibitor, or a true difference in the interactions of the MPR and fusion loops in the prefusion and postfusion states that is relevant to understanding the mechanism of membrane fusion by herpesvirus gBs.

### The bound fusion inhibitor

The importance of the interprotomer interactions between the fusion loops, the MPR, and the TM in maintaining the prefusion conformation is confirmed by their reinforcement by the potent HCMV-specific fusion inhibitor WAY-174865 (*18*–*20*), which is clearly defined in the prefusion gB mass density (fig. S3) and binds all three of these structural elements (Figs. 1B and 3, F and G, and fig. S6). The interactions between WAY-174865 and gB are mainly hydrophobic and aromatic, involving multiple hydrophobic residues, including Y153 and Y155 in the fusion loop; L712, L715, I730, A738, and F747 in the MPR; and P751 and F752 in the TM (Fig. 3F). WAY-174865 also forms multiple hydrogen bonds with gB. The amide carbonyl in WAY-174865 forms hydrogen bond with the backbone Cα-H of L712. The CF3 disubstituted phenyl ring and the side-chain carbonyl group of N750 form an electrostatically driven carbonyl-*p* aromatic interaction (Fig. 3G) (*29*).

Previously observed structure-activity relationships for the family of CMV fusion inhibitors can be better understood based on the cryo-EM structure. For example, the chiral (*S*)-methyl group has a profound effect on the anti-CMV activity: WAY-174865 is 3-fold more potent than the R-enantiomer, 10-fold more potent than the desmethyl analog, and 30-fold more potent than the inhibitor without the methylene linker (*20*). The improved potency can be attributed to the preferable L-shape inhibitor conformation that is restricted by the chiral (*S*)-methyl group (*30*). This inhibitor conformation better fits the curvature of the binding site and forms an interprotomer “hook” that reinforces the interprotomer ring of MPR α9 helices around the threefold axis (Figs. 3, F and G, and 4C). Substitutions on the middle phenyl ring of this class of inhibitors mostly decrease potency (*19*). Consistent with this finding, the ring is enclosed in a relatively tight space that may not accommodate additional groups.

WAY-174865 inhibits CMV infectivity with a 50% inhibitory concentration of 0.6 nM and a selectivity index of at least ~10,000-fold against HSV and varicella-zoster virus (VZV) (*31*). Residues of CMV gB that interact with the CF3 disubstituted phenyl ring are conserved between these viruses, but the CMV residues that interact with the remainder of the inhibitor are not shared with HSV or VZV, explaining the selectivity of virus inhibition (fig. S6). Inhibitor escape mutations are also explained by the structure. HCMV strain AD169 escapes inhibition through mutation of residues homologous to strain Towne G734 and F752 (*18*), which are WAY-174865 binding residues on α9 of the MPR and α10 of the TM, respectively (Fig. 3F).

### Correspondence of the gB antigenic map to the prefusion gB structure

**Fig. 5:**
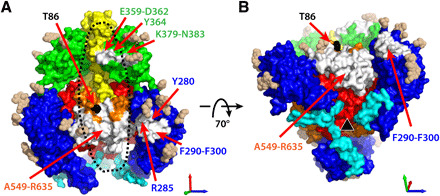
Antigenic surfaces and potential gH/gL binding site on the prefusion gB ectodomain (residues 86 to 721). (**A**) View perpendicular to the threefold axis. (**B**) Oblique view from below the viral envelope. The model is rotated 70° as indicated around the black vector perpendicular to the threefold axis. Domains are colored as in Fig. 1A; glycans are beige. Surfaces from residues that bind anti-gB complement-independent neutralizing antibodies are white and labeled in orange type for AD1, green type for AD4, and blue type for AD5. Residues in AD1 were identified by peptide mapping; residues in AD4 and AD5 were identified by x-ray crystal structures of FAb-antigen complexes and mAb neutralization escape mutations (references provided in table S2). The most N-terminal residue in buildable density, T86, is black. Approximate gH/gL binding site (fig. S8) based on published cryo-EM image of CMV virion (*6*) is indicated with black dashed circle. Threefold axis is indicated with a black triangle.

CMV gB is a prominent target of antibodies elicited by infection. Although some neutralize CMV infectivity on both fibroblasts and epithelial cells, inhibiting post-attachment entry events, many antibodies that bind gB are non-neutralizing, and others require complement for their antiviral activity (*32*–*34*). Five gB antigenic domains (AD-1 to AD-5) have been identified. AD-3 corresponds to the cytoplasmic tail and is not the target of neutralizing antibodies (table S2). AD-5, on structural domain I, is an exposed target of neutralizing antibodies (*7*, *35*) and has the same local structure in prefusion and postfusion gB (Figs. 2 and 5 and table S2).

AD-4, on structural domain II (*21*, *36*, *37*), contains the binding site of the broadly neutralizing mAb SM5-1, the FAb of which we used to immunoaffinity purify gB from CMV virions (Figs. 2 and 5, fig. S7, and table S2). The cryo-EM image reconstructions of complexes of the SM5-1 FAb in complex prefusion or postfusion gB demonstrate that it binds both of these gB conformations (figs. S2C and S7A). The previously determined crystal structure of an isolated HCMV gB domain II in complex with the SM5-1 FAb [Protein Data Bank (PDB) accession code 4OT1] (*21*) fits the cryo-EM density of postfusion gB in complex with the FAb well (fig. S7B). A shift in the binding angle of the FAb to domain II of the prefusion structure is needed to relieve modeled steric hindrance between the FAb and residues in domain III (fig. S7, C and D). Although the SM5-1 FAb neutralizes CMV (*21*), reduction in the prefusion content of FAb-gB complex preparations over time demonstrates that the bound FAb does not prevent the prefusion to postfusion rearrangement.

AD-1, on structural domain IV, has been roughly mapped to residues 549 to 635 by antibody binding to synthetic peptides and recombinant protein constructs but has not been precisely defined by high-resolution FAb-antigen complex structures or neutralization escape mutations (table S2) (*38*, *39*). In postfusion gB, AD-1 is part of the prominent, membrane-distal domain IV knob, which is highly accessible to antibodies, although some residues of the peptides that bind AD-1 neutralizing mAbs are buried in the trimeric interface (*7*). In prefusion gB, domain IV is part of the inner core of the molecule, with much of its surface covered by domains I, II, and V, but with an antibody-accessible surface, largely devoid of glycans, at the base of a large oval-shaped hollow between the “tripod legs” (Fig. 5A).

The last antigenic domain, AD-2, is not resolved in either the prefusion or postfusion gB structure. It is part of the flexible N-terminal 85 residues, which contain the conserved site I (residues 68 to 77) linear epitope, which is the target of mAbs that require bivalency to neutralize (table S2) (*33*). The connection of AD-2 to the resolved structure of prefusion gB at T86 is adjacent to the exposed surface of domain IV at the base of the hollow between the tripod legs (Fig. 5).

The epitopes of complement-independent neutralizing mAbs occupy the base and rim of the hollow in prefusion gB (Fig. 5). Consistent with biochemical evidence that interaction between gB and gH/gL may have a role in triggering gB-mediated cell entry (*26*, *28*), cryo-ET of CMV virions shows an L-shaped density, attributed to gH/gL, contacting molecules on the virion surface that have the approximate shape of prefusion gB (*6*). The structure of gH/gL in the pentameric complex (PDB access code 5VOB) (*5*), when modeled in contact with prefusion gB based on published images of the cryo-ET density (*6*), is compatible with gH binding gB in the glycan-free hollow (fig. S8). These findings suggest that the N-terminal flexible region that contains AD-2 may bind gH/gL, and complement-independent neutralizing antibodies that bind AD-2 may block gH/gL–prefusion gB interactions.

Like postfusion gB, prefusion gB also has a protrusion at its membrane-distal end (fig. S5A). Unlike the well-defined domain IV density in the postfusion protrusion, the mass density for the furin-cleaved junction between domains II and III in the prefusion gB protrusion is too poorly defined for model building and has too small a volume (3000 Å^3^ at a ~10 σ threshold) to accommodate the 46–amino acid residues and four oligosaccharides per protomer in the unbuildable region between Q436 and Q483 (estimated to be ~40,000 Å^3^, assuming a single protease cleavage). Also, unlike the postfusion gB protrusion, which is the target of many AD-1 antibodies (table S2), no antibody binding sites have been mapped to the prefusion gB protrusion, which has many of the hallmarks of an immunodominant site on a viral glycoprotein—high accessibility to antibodies, high variability between strains, glycan shielding, and flexibility (fig. S5B). The prefusion gB protrusion likely rearranges in the transition to the extended intermediate, as it contains the hinges between the gB central core and the domain I/II units that swing up to attack the host cell membrane. This rearrangement makes the protrusion a potential binding site for rearrangement-inhibiting antibodies and a site that may not have been identified in experiments that use postfusion gB as the target antigen for mAb-screening strategies.

### Comparison to VSV prefusion G

Structures that correspond to much of domain V, the MPR, and the TM of prefusion CMV gB are absent from the determined prefusion VSV G structure, preventing comparisons of these elements between the distant homologs (*13*). For the domains that are in buildable density for both structures, the overall tripod structural arrangement and some elements of the prefusion to postfusion rearrangement are similar. However, prefusion CMV gB has distinctive features. For example, prefusion CMV gB has far more extensive interprotomer interfaces (~11,200 Å^2^ per protomer) and much greater lengths of coiled coil on the threefold axis than VSV G, which only buries 1600 Å^2^ per protomer in observed interfaces (although there could be additional unobserved interfaces) and with little contribution from a central ectodomain coiled coil. During rearrangement, the VSV G central coiled coil in the equivalent to domain III extends (*13*); the much longer equivalent coiled coil in gB does not extend (at least not within buildable density). The determined elements of the VSV G trimer are thought to transiently dissociate during the prefusion to postfusion rearrangement (*13*, *40*). For CMV gB, this dissociation is not necessary topologically and is very unlikely, given the extent of coiled coil interactions around the threefold axis of the ectodomain. VSV G is in a pH-dependent, dynamic equilibrium between prefusion and postfusion states (*41*); CMV gB transitions irreversibly from prefusion to postfusion. Accordingly, a cluster of histidines in prefusion VSV G at the equivalent of the interface of CMV gB domain I with domain IV and an N-terminal part of domain V provides low-pH destabilization of prefusion VSV G, and acidic residues brought together in postfusion G provide a high-pH destabilization of that conformation (*12*). Such pH-sensitive “switches” are absent from the prefusion and postfusion gB structures.

## DISCUSSION

We determined a structure of virion-derived, cross-linked, fusion inhibitor– and antibody fragment–bound CMV gB in a conformation that is different from the previously determined postfusion structure (*7*, *8*, *10*, *11*). The structural model fits well within the lower-resolution cryo-ET density on HCMV virions that has been attributed to prefusion gB (*6*); the fusion loops are sequestered by interaction with other parts of the molecule, as is the case for other prefusion viral glycoproteins (*27*); and the molecule is bound by a fusion inhibitor and explains the inhibitor’s mechanism of action, structure-activity relationships, and pattern of inhibition escape mutations (*18*–*20*). Thus, our high-resolution structure represents the prefusion conformation of HCMV gB. The structure demonstrates a basic architecture in the pattern of the prefusion conformation of VSV G (*13*) but with differences indicating greater trimer stability consistent with its irreversible rearrangement during cell entry. The determination of the structure of the entirety of domain V, the MPR, and most of the TM domain has revealed the mode of interaction of the MPR with the viral membrane, the sheathing of the hydrophobic fusion loops by the MPR, and the rearrangement of domain V that drives the transition from an extended intermediate form to the postfusion form. Mapping neutralizing epitopes onto the surface of the molecule reinforces the hypothesis that interference with gH/gL binding is a potential mechanism of neutralization. The visualization of a fusion inhibitor bound in the interface between the fusion loops, MPRs, and proximal TM domain shows how the inhibitor stabilizes the prefusion conformation and provides high-resolution data that can guide structure-based drug design. Limitations of the study include the unresolved AD-2 and the unbuildable density of the membrane-distal junction between domains II and III.

A key goal of CMV research is the design of a prophylactic CMV vaccine to prevent the lifelong disability associated with congenital CMV disease. On the basis of analogy to RSV vaccine candidates (*9*), we hypothesize that prefusion gB may prove more effective than postfusion gB at eliciting neutralizing antibodies. Achieving sufficient conformational stability of prefusion gB for structure determination required the formation of a reversible complex with an inhibitor, making the stabilized molecule unsuitable as a vaccine antigen—the inhibitor would likely diffuse away after injection, leading to loss of the prefusion conformation. The structure provides the high-resolution data needed to design inhibitor-free prefusion gB immunogens that can be used to test the hypothesis that prefusion gB is a superior vaccine antigen and a key component of a safe and effective congenital CMV vaccine.

## MATERIALS AND METHODS

### Isolation and purification of cross-linked and native HCMV gB with fusion inhibitor

Minor variations on the following protocol were used to prepare lots of prefusion HCMV gB used for structure determination. Human foreskin fibroblast cells in roller bottle culture in Dulbecco’s modified Eagle’s medium with 15% heat-inactivated fetal bovine serum were inoculated with HCMV strain Towne at a multiplicity of infection of 0.01 (based on titration in MRC-5 cells) and incubated at 37°C for 12 days, when nearly complete cytopathic effect was observed. The remaining attached cells were scraped from the roller bottles, and the cell culture medium was harvested, frozen and thawed, sonicated, and clarified by low-speed centrifugation. WAY-174865 (*18*, *19*) was added to 1 mg/liter before the harvest was concentrated by tangential flow filtration with a 300-kDa cutoff membrane, and the fusion inhibitor was maintained at this concentration or higher throughout all subsequent steps. The concentrated virus was frozen, thawed, and pelleted twice through 15% sucrose in phosphate-buffered saline (PBS) onto 40% iodixanol cushions. The concentrated virus was cross-linked with 0.2 mM bis(sulfosuccinimidyl) glutarate-d0 (Thermo Fisher Scientific) at room temperature for 1 hour before quenching with 5 mM tris. The purified and cross-linked virus was lysed with 2% *n*-dodecyl β-d-maltoside (DDM) in the presence of WAY-174865 (2 mg/liter). The solubilized material was clarified by high-speed centrifugation, concentrated by ultrafiltration with an Ultracel-15 Amicon 100-kDa membrane, and incubated overnight at 4°C with a FAb of mAb SM5-1 (*21*) that had been fused at the C terminus to a histidine tag and a Strep-tag (LakePharma). The FAb-bound protein was purified by affinity chromatography with a HiTrap TALON cobalt column (GE Healthcare) with imidazole elution and addition of EDTA to 10 mM following elution. After concentration with a 100-kDa ultrafiltration membrane, FAb-bound gB was further purified by size exclusion chromatography with a Superose 6 10/300 GL (GE Healthcare) column in PBS (pH 7.4), 0.1% DDM, and WAY-174865 (2 mg/liter). Pooled fractions were concentrated by ultrafiltration as before, and the FAb-bound gB was further purified by affinity chromatography with a StrepTrap HP Purification column (GE Healthcare) in the same buffer, with elution by 2.5 mM desthiobiotin. The purified gB-FAb complex was concentrated by ultrafiltration as above and dialyzed against PBS, 0.1% DDM, 1 mM EDTA, and WAY-174865 (2 mg/liter) before being used for cryo-EM grid preparations.

### Electron microscopy

Aliquots of purified cross-linked gB-FAb complexes (0.8 mg/ml) were vitrified on graphene oxide film–supported EM grids (*42*) using a Vitrobot (Thermo Fisher Scientific). The frozen grids were transferred to a FEI Titan Krios transmission electron microscope that operates at 300 kV for screening and data collections with SerialEM (*43*) at a nominal magnification (×18,000) using a K2 direct detector camera (Gatan) with super-resolution movie mode. The unbinned pixel size was 0.688 Å, and the beam intensity was ~8e per unbinned pixel per second. The total electron dose on the sample for each movie was ~40e/Å^2^. A total of 7768 movies, each with 28 frames, was collected in three sessions.

### Image processing

Drift correction was done using the MotionCor2 (*44*), and the final micrographs were binned 2× and averaged from all frames. Contrast transfer function parameters were calculated with Gctf (*45*). For particle picking, the published structure of HCMV gB in the postfusion conformation [PDB: 5CXF; (*8*)] was used to generate a 30-Å density map using pdb2mrc [EMAN; (*46*)]. Projection images from this density map were generated with project3d (EMAN) and used as a template for the automatic particle picking using Gautomatch (*47*). Relion v2.1-beta (*48*) was used to extract the resulting ~1.9 million particles and to carry out all subsequent image processing steps, including 2D classification, 3D classification, auto-refinement, and postprocessing.

The 2D classes were put into three groups based on the image features: The first group consisted of the 2D classes that showed features that resemble the crystallographically determined postfusion gB structure (~55%), the second group contained 2D classes with well-resolved protein features that do not resemble the structural features from postfusion gB (~13%), and the third group contained 2D classes that did not contain clearly defined protein (~32%). The first and second groups were further processed with 3D classification, auto-refinement, and postprocessing procedures with Relion, during which C3 symmetry was applied. Following this processing, a ~3.5-Å-resolution electron density map showing the postfusion conformation structure was reconstructed from the first group, and a ~3.6-Å-resolution electron density map showing a prefusion conformation structure was reconstructed from the second group (fig. S2A). On the basis of these density maps and the known HCMV gB amino acid sequence (Towne strain UniProtKB: P13201), atomic models of the prefusion and postfusion structures were built with Coot (*49*). To build the prefusion conformation model, domains I, II, III, and IV from the reference PDB:5CXF model were docked as rigid bodies into the electron density map as a starting point. Then, adjustments of individual residues were made for optimal fitting. The models for domains V, MPR, and TM were built de novo. The models were iteratively refined with the Phenix.real_space_refine tool (*50*) followed by local manual adjustment for several rounds. The model figures were generated with the PyMOL Molecular Graphics System version 2.0 (Schrödinger LLC) and Chimera (*51*).

### Homology modeling and analysis of inhibitor binding environment

Homology models of gB structures of HCMV (AD169), HSV (Patton), and VZV (Ellen) were generated with MOE (*52*). The residues at equivalent locations were identified manually from comparison of these homology models.

### Sequence alignment and glycosylation site prediction

Fifty HCMV gB protein sequences from Genbank and an in-house database including amino acids 436 to 483 (positions based on gB from Towne strain) were extracted using in-house Perl scripts in FASTA format. The multiple sequence alignment was performed with ClustalW (*53*), and the graphical representation of the amino acid multiple sequence alignment was generated by Weblogo in sequence logo format (*54*, *55*).

## SUPPLEMENTARY MATERIALS

Supplementary material for this article is available at http://advances.sciencemag.org/cgi/content/full/7/10/eabf3178/DC1

## Appendix

View/request a protocol for this paper from *Bio-protocol*.
